# Supplementary material for: Coherent theta activity in the medial and orbital frontal cortices encodes reward value
Source: eLife. 2021 Sep 10;10:e63372. doi: 10.7554/eLife.63372 (PMC8457826; doi:10.7554/eLife.63372)
Supplement: Transparent reporting form [file elife-63372-transrepform1.docx]

***eLife’s* transparent reporting form**

We encourage authors to provide detailed information *within their submission* to facilitate the interpretation and replication of experiments. Authors can upload supporting documentation to indicate the use of appropriate reporting guidelines for health-related research (see [EQUATOR Network](about:blank)), life science research (see the [BioSharing Information Resource](about:blank)), or the [ARRIVE guidelines](about:blank) for reporting work involving animal research. Where applicable, authors should refer to any relevant reporting standards documents in this form.

If you have any questions, please consult our Journal Policies and/or contact us: [editorial@elifesciences.org](about:blank).

**Sample-size estimation**

- You should state whether an appropriate sample size was computed when the study was being designed
- You should state the statistical method of sample size computation and any required assumptions
- If no explicit power analysis was used, you should describe how you decided what sample (replicate) size (number) to use

Please outline where this information can be found within the submission (e.g., sections or figure legends), or explain why this information doesn’t apply to your submission:

The sample size was determined based on prior multi-electrode arrays studies. The number of animals that were used is comparable to previous studies from our lab (e.g., Amarante et al., 2017, J Neurosci). For experiment 1, 12 rats were initially trained to perform the behavioral task and neural recordings were obtained from 10 of these animals for the medial frontal cortex and from 6 of these animals for the orbital frontal cortex. The same group of 12 rats was used for experiments 2 and 3. All viable recordings of LFP activity, from 16 electrodes implanted in each brain are was included in study, after screening for unstable signals or prominent peaks 157 at 60 Hz in plots of power spectral density. This information is specified in the Methods section and Results sections for each experiment in the manuscript.

**Replicates**

- You should report how often each experiment was performed
- You should include a definition of biological versus technical replication
- The data obtained should be provided and sufficient information should be provided to indicate the number of independent biological and/or technical replicates
- If you encountered any outliers, you should describe how these were handled
- Criteria for exclusion/inclusion of data should be clearly stated
- High-throughput sequence data should be uploaded before submission, with a private link for reviewers provided (these are available from both GEO and ArrayExpress)

Please outline where this information can be found within the submission (e.g., sections or figure legends), or explain why this information doesn’t apply to your submission:

Each data set (a recording of local field potential, or LFP) was obtained from one of the 12 rats used in the study. Each data sample was from a slightly different location (relative to standard rodent atlas landmarks and cortical laminae). All statistical analysis such as t-tests and ANOVAs included a factor for the rat from which the data was collected. No gross outliers (defined using methods for Exploratory Data Analysis were detected, behaviorally or electrophysiologically).

**Statistical reporting**

- Statistical analysis methods should be described and justified
- Raw data should be presented in figures whenever informative to do so (typically when N per group is less than 10)
- For each experiment, you should identify the statistical tests used, exact values of N, definitions of center, methods of multiple test correction, and dispersion and precision measures (e.g., mean, median, SD, SEM, confidence intervals; and, for the major substantive results, a measure of effect size (e.g., Pearson's r, Cohen's d)
- Report exact p-values wherever possible alongside the summary statistics and 95% confidence intervals. These should be reported for all key questions and not only when the p-value is less than 0.05.

Please outline where this information can be found within the submission (e.g., sections or figure legends), or explain why this information doesn’t apply to your submission:

All relevant information on the statistical analysis, animal and LFP data sizes, measures of central tendency and spread, statistical measures, and p values are included in the manuscript. p values were reported to the third decimal place.

(For large datasets, or papers with a very large number of statistical tests, you may upload a single table file with tests, Ns, etc., with reference to sections in the manuscript.)

**Group allocation**

- Indicate how samples were allocated into experimental groups (in the case of clinical studies, please specify allocation to treatment method); if randomization was used, please also state if restricted randomization was applied
- Indicate if masking was used during group allocation, data collection and/or data analysis

Please outline where this information can be found within the submission (e.g., sections or figure legends), or explain why this information doesn’t apply to your submission:

Grouping was done for recordings in either the medial or orbital frontal cortex, based on the placement of recording arrays in these regions and post mortem histological confirmation of probe placement. No other treatments were done in the study.

**Additional data files (“source data”)**

- We encourage you to upload relevant additional data files, such as numerical data that are represented as a graph in a figure, or as a summary table
- Where provided, these should be in the most useful format, and they can be uploaded as “Source data” files linked to a main figure or table
- Include model definition files including the full list of parameters used
- Include code used for data analysis (e.g., R, MatLab)
- Avoid stating that data files are “available upon request”

Please indicate the figures or tables for which source data files have been provided:

Code, examples of raw data, and summaries of grouped data will be made available via OSF if the manuscript is accepted for publication. In the meantime, they are available by request from the corresponding author.
